# Supplementary material for: Relationship between Changes in Intestinal Microorganisms and Effect of High Temperature on the Growth and Development of Bombyx mori Larvae
Source: Int J Mol Sci. 2022 Sep 7;23(18):10289. doi: 10.3390/ijms231810289 (PMC9499401; doi:10.3390/ijms231810289)
Supplement: Supplementary file 1 [file ijms-23-10289-s001.zip › ijms-1847372-supplementary.pdf]

Relationship between changes in intestinal microorganisms and effect of high temperature on the growth and development of *Bombyx mori* larvae

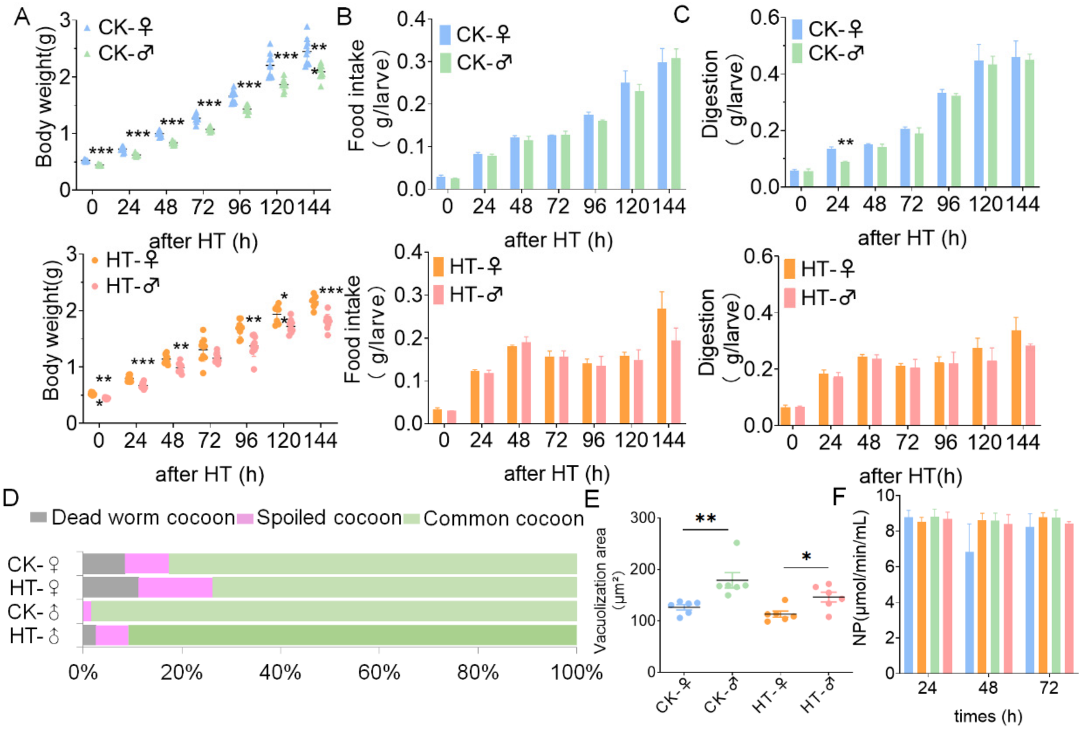

Figure S1. Investigation on growth and physiological indexes of silkworm. (A) Investigation of silkworm weight, n ≥ 7. (B) Food intake capacity of the larvae (grams per larva). (C) Digestion capacity of the larvae (grams per larva). (D) The statistical results of the number of dead cages, ordinary cocoons, and crumb cocoons, N=3, n =11-24. (E) Quantitative results of vacuolation of midgut tissue cells in the CK group and HT group (72 h). (F) Protease activity in digestive juice during high-temperature treatment. *, P≤ 0.05; **, P≤ 0.01; ***, P≤ 0.001. CK, control group; after HT, high-temperature treatment.

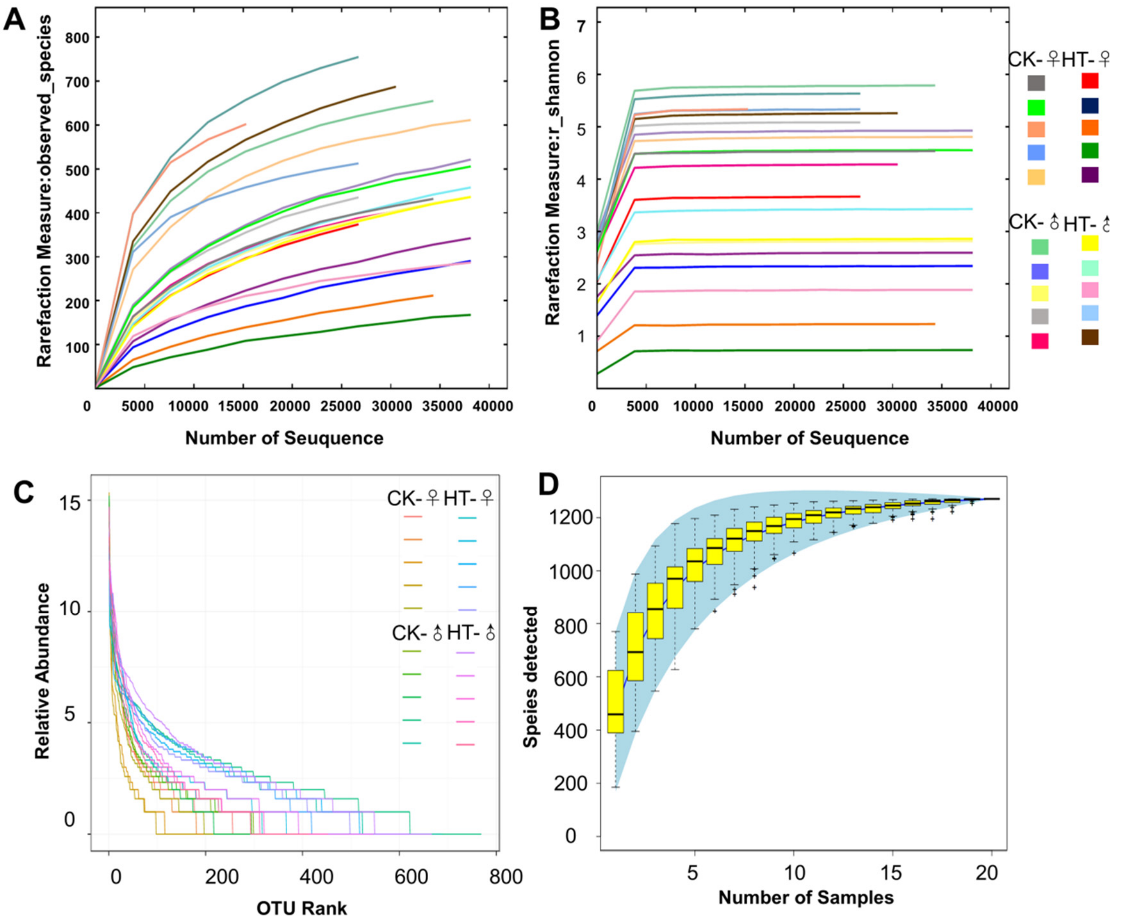

Figure S2. Alpha Diversity analysis of silkworm gut microbiota. (A) Rarefaction curve of samples. Each curve represents a sample, when the curve tends to be gentled, it indicates that the amount of sequencing data at this time is reasonable. (B) Shannon-wiener of samples. Each curve represents a sample, the initial curve straight climb is due to the number of sequencing is far from covering the samples, the value rises until the smooth description shows that the number of sequencing is sufficient to cover most of the microbiota in the samples. (C) Rank-abundance of samples. Each curve represents a sample, abscissa indicates species abundance, the ordinate indicates the proportion of the abundance of the species (the log2 value), the wider the curve on the horizontal axis, the richer the composition of the species. (D) Species accumulation curves of samples. Applicable to the same type of sample number> 10, the curve tends to mean that the species in this environment will not increase significantly with the increase in samples size.

Table S1. Alpha diversity index calculation results.

Samples	Valid reads	OTU	Chao1	ACE	Simpson	Shannon
NT-♀1	37494	1978	276	418.9323	0.9150	4.4708
NT-♀2	41011	2304	305	420.0586	0.9044	4.4765
NT-♀3	21463	2798	517	517.0000	0.8300	5.2810
NT-♀4	30281	2295	418	450.3528	0.9155	5.2936
NT-♀5	42607	2759	425	502.2482	0.8821	4.7307
Average	34571.2	2426.8	388.2	461.7184	0.8894	4.8505
NT-♂1	38747	2953	493	614.6606	0.9498	5.7269
NT-♂2	45761	2449	310	464.5643	0.9337	4.8480
NT-♂3	42799	2018	236	322.8468	0.5863	2.7219
NT-♂4	30367	2037	311	516.7521	0.9446	5.0364
NT-♂5	33061	1836	288	409.7647	0.8684	4.2315
Average	38147	2258.6	327.6	465.7177	0.8566	4.5129
HT-♀1	29972	1760	255	439.1805	0.8204	3.6102

HT-♀2	48807	1488	130	190.7999	0.5576	2.2694
HT-♀3	36308	968	100	174.6254	0.2951	1.1745
HT-♀4	45913	833	72	120.8025	0.1567	0.6864
HT-♀5	38527	1563	162	259.2756	0.6929	2.5172
Average	39905.4	1322.4	143.8	236.9368	0.5045	2.0515
HT-♂1	40567	1998	220	382.0341	0.6633	2.7595
HT-♂2	38575	2055	247	372.3733	0.7656	3.3434
HT-♂3	47780	1400	171	215.7520	0.3671	1.8287
HT-♂4	30821	3360	621	767.5486	0.9098	5.5862
HT-♂5	31720	3030	523	678.3814	0.9171	5.1966
Average	37892.6	2368.6	356.4	483.2179	0.7246	3.7429
